# Ferroptosis Induction by Fenbendazole Combined With Photothermal Therapy Triggers Dual‐Immunotherapy Against Bladder Cancer

**DOI:** 10.1002/advs.74876

**Published:** 2026-03-20

**Authors:** Xiaojian Xu, Rui Liang, Anguo Zhao, Jun Zhang, Rongkang Li, Yang Liu, Dashi Deng, Qi Zhuang, Lisha Liu, Lei Peng, Miao Li, Xuedong Wei, Yuhua Huang, Shaohua Zhang, Liang Cheng, Jianquan Hou

**Affiliations:** ^1^ Department of Urology The First Affiliated Hospital of Soochow University Suzhou China; ^2^ Department of Urology The Fourth Affiliated Hospital of Soochow University Medical Center of Soochow University Suzhou Dushu Lake Hospital Suzhou China; ^3^ Department of Urology South China Hospital Medical School Shenzhen University Shenzhen China; ^4^ Department of Urology The Affiliated Luohu Hospital of Shenzhen University Shenzhen University Shenzhen China; ^5^ Institute of Functional Nano & Soft Materials (FUNSOM) Jiangsu Key Laboratory for Carbon Based Functional Materials & Devices Soochow University Shenzhen China

**Keywords:** bladder cancer, ferroptosis, flubendazole, photothermal therapy, polydopamine

## Abstract

Ferroptosis, a newly recognized form of regulated cell death, has emerged as a promising strategy in cancer therapy. Given the high incidence and recurrence rates of bladder cancer, exploring novel therapeutic approaches is critically important. In this study, we developed a novel intravesical nanoplatform, FBZ@BSA@PDA, that integrates ferroptosis‐related oxidative injury immunogenic cell death (ICD) activation, and photothermal therapy (PTT) to achieve synergistic treatment of bladder cancer. The hydrophobic drug fenbendazole (FBZ) was efficiently encapsulated via thermally induced unfolding of bovine serum albumin (BSA), and polydopamine (PDA) was formed by in situ polymerization of dopamine to enhance tissue adhesion and photothermal responsiveness. Following transurethral intravesical administration, the platform enabled sustained drug release and localized PTT. Mechanistically, it induced lipid peroxidation (LPO), GSH depletion, and mitochondrial dysfunction, which suggest that ferroptosis may contribute to tumor cell death. This was accompanied by key ICD markers, including calreticulin (CRT) exposure, high mobility group box 1 (HMGB1) release, and adenosine triphosphate (ATP) secretion, which effectively promoted dendritic cell (DC) maturation and T‐cell activation. FBZ@BSA@PDA demonstrated strong anti‐tumor efficacy and favorable biosafety in an orthotopic mouse model of bladder cancer. This strategy offers a promising localized immune‐potentiated therapeutic approach for clinical bladder cancer treatment.

## Graphical abstract

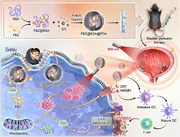

## 1 Introduction

Malignant tumors, major global public health issues, have attracted significant attention from researchers worldwide [1]. Among them, bladder cancer is the most common malignancy of the urinary system [2]. Owing to their high recurrence rate, drug resistance, and tendency to metastasize, traditional treatment methods often have limited efficacy [3, 4]. The bladder, as a special type of hollow organ, has unique therapeutic approaches, such as transurethral resection of bladder tumor (TURBT) and postoperative intravesical chemotherapy [5, 6]. Intravesical therapy has gained considerable attention for its advantages, including localized drug delivery, high local drug concentrations, and relatively few systemic side effects [7]. Currently, commonly used intravesical agents include chemotherapeutic drugs such as gemcitabine and epirubicin, as well as immunotherapeutic agents such as Bacillus Calmette–Guérin (BCG), which have been shown to be effective in preventing recurrence [8, 9]. However, the limitations of intravesical therapy, such as noticeable side effects, drug resistance, and limited efficacy in preventing recurrence, highlight the urgent need to develop novel and more effective intravesical treatment strategies for bladder cancer [10, 11, 12].

Ferroptosis is a novel form of cell death distinct from apoptosis and necrosis, and it holds significant implications in cancer research [13]. Several ferroptosis inducers, such as erastin and sorafenib, have been shown to effectively trigger ferroptosis, offering new approaches for cancer therapy [14, 15, 16]. Photothermal therapy (PTT) achieves local tumor destruction by relying on thermal stress and reactive oxygen species (ROS) generation [17, 18]. This therapy holds great promise, particularly for bladder cancer which is a unique hollow organ. However, in the acidic tumor microenvironment (TME), a reduction in pH inhibits the Fenton or Fenton‐like reactions, which subsequently impedes the effective generation of hydroxyl radicals (·OH). PTT, by locally incresing the temperature, can significantly accelerate the production of ·OH, thereby enhancing ROS accumulation within tumor cells and promoting the occurrence of ferroptosis [19, 20]. Both ferroptosis and PTT can induce immunogenic cell death (ICD) [21, 22], which activates T‐cell immune responses and further boosts anti‐tumor immunity [23, 24]. Therefore, integrating the synergistic mechanisms of ferroptosis, PTT, and ICD may help develop an efficient and precise anti‐tumor treatment strategy.

**FIGURE 1:**
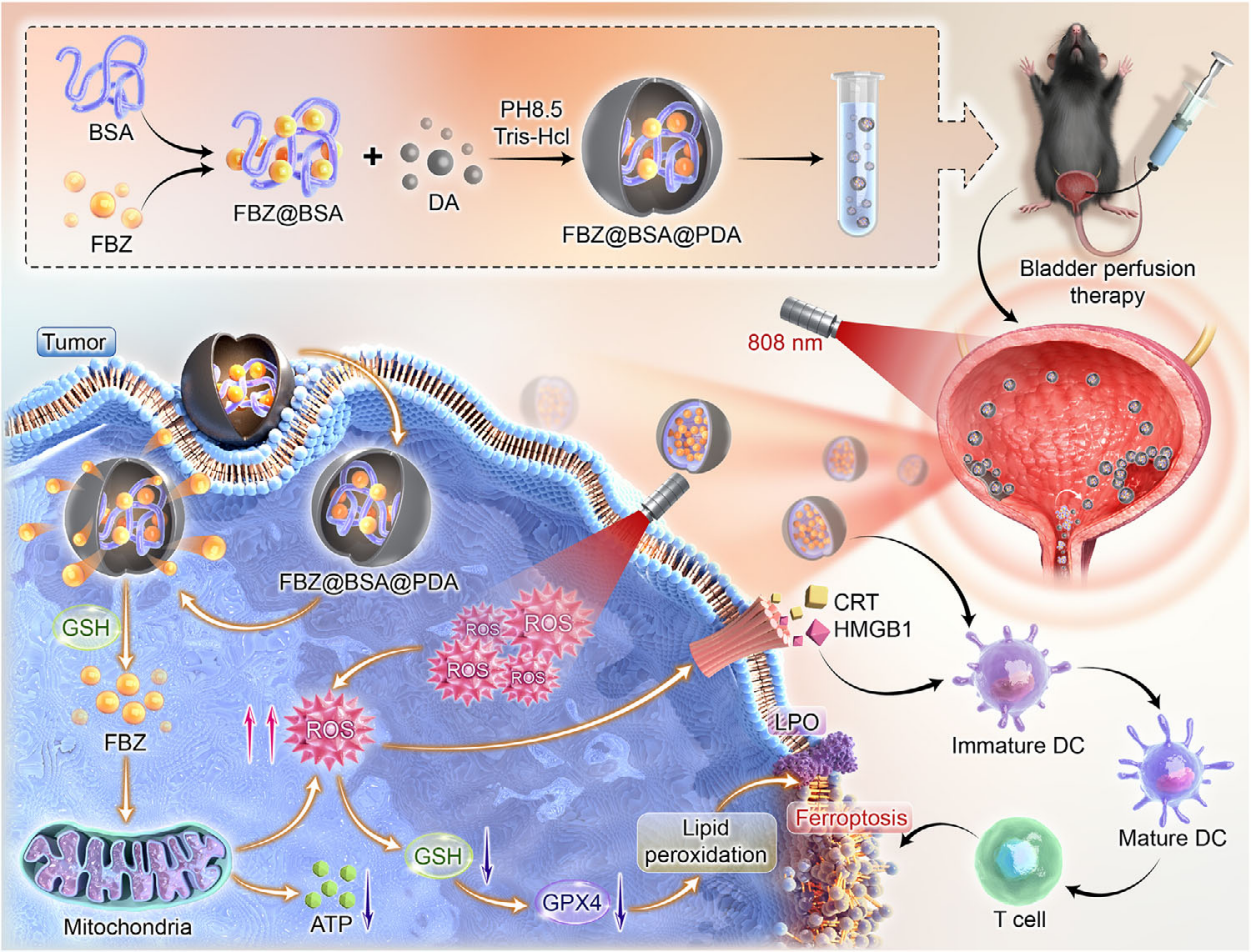
The novel intravesical delivery system exhibits GSH‐responsive drug release, enabling a synergistic therapeutic strategy that combines ferroptosis, ICD and PTT. Upon near‐infrared (NIR) laser irradiation, the system facilitates FBZ release and significantly elevates ROS levels, thereby initiating ferroptosis. The combined effects of ferroptosis and photothermal stimulation further promote ICD, effectively activating anti‐tumor immune responses and enhancing therapeutic efficacy.

To address the current limitations of drug perfusion therapies, we have chosen a novel drug with anti‐tumor properties, flubendazole (FBZ), as the core therapeutic agent for bladder perfusion treatment. FBZ exhibits multiple anti‐tumor characteristics, including potential ferroptosis‐inducing activity, as well as immune activation potential [25]. It can promote dendritic cell (DC) maturation, leading to immune‐mediated tumor cell death [26]. However, owing to its poor solubility in water, FBZ has not yet been utilized in anti‐tumor research. To improve the water solubility of FBZ and achieve synergistic enhancement with photothermal therapy, we encapsulated the small‐molecule drug in bovine serum albumin (BSA) and introduced polydopamine (PDA)—a material with strong adhesiveness and excellent photothermal properties—via in situ polymerization. Based on our previously established synthesis strategy [26, 27], we ultimately constructed a novel intravesical drug delivery platform, FBZ@BSA@PDA, designed to enable photothermal‐enhanced nanocatalysis and immune‐modulatory synergistic antitumor therapy. This platform was designed for photothermal‐enhanced nanocatalysis and immune‐modulatory synergistic therapy against tumors (Figure 1). We assessed the cytotoxicity of FBZ@BSA@PDA on bladder cancer cells in mice and its ability to activate immune responses in vitro. Furthermore, we developed an orthotopic mouse bladder cancer model to evaluate the anti‐tumor potential of FBZ@BSA@PDA. It could be found that FBZ@BSA@PDA effectively killed tumor cells and promoted the maturation of DC. Additionally, bladder perfusion with this platform provided a potent and efficient treatment for bladder cancer. These findings suggested that FBZ@BSA@PDA held significant promise as a novel therapeutic approach for bladder cancer.

## 2 Results and Discussion

### 2.1 Preparation and Characterization of FBZ@BSA@PDA Nanoparticles

**FIGURE 2:**
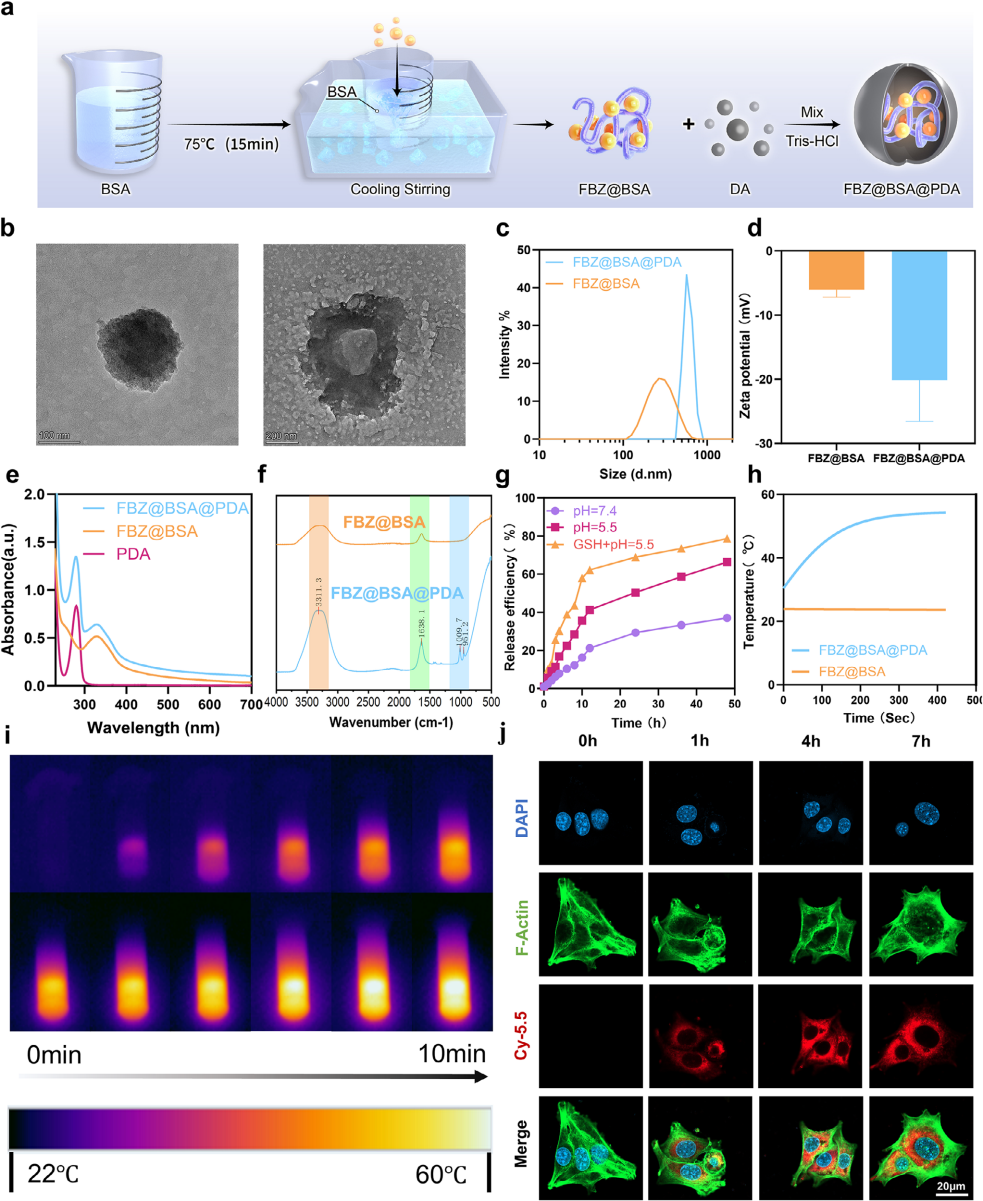
Preparation and characterization of FBZ@BSA@PDA. a) Schematic illustration of the synthetic procedure for FBZ@BSA@PDA. b) TEM images of FBZ@BSA and FBZ@BSA@PDA. c, d) Size distributions and zeta potentials (n = 3) of FBZ@BSA and FBZ@BSA@PDA. e) UV–vis absorption spectra of PDA, FBZ@BSA, and FBZ@BSA@PDA. f) FT‐IR spectra of FBZ@BSA and FBZ@BSA@PDA. g) Cumulative drug release profiles of FBZ@BSA@PDA under different conditions. h) Temperature elevation curves of FBZ@BSA and FBZ@BSA@PDA under 808 nm laser irradiation (1.5 W/cm^2^). i) Infrared thermal imaging of the photothermal performance of FBZ@BSA@PDA. j) CLSM images showing the uptake of FBZ@BSA@PDA@Cy5.5 by MB49 cells after 1, 4, and 7 h of co‐incubation. The cell nuclei were stained with 4′,6‐diamidino‐2‐phenylindole (DAPI, blue), and the F‐actin filaments were stained with phalloidin (green). Scale bar=10 µm.

Building upon our previous work, FBZ was mixed with BSA under mild thermal annealing (≈75 °C) to form the FBZ@BSA complex (Figure 2A). Subsequently, the FBZ@BSA complex was added to a dopamine hydrochloride solution and incubated at 37 °C to simulate the in‐situ polymerization process within the bladder cavity, resulting in the formation of FBZ@BSA@PDA nanoparticles. We further examined the morphology of the nanoparticles using transmission electron microscopy (TEM) (Figure 2b; Figure S1). The FBZ@BSA particles appeared as dispersed oval‐shaped nanoparticles with a primary size of ∼ 267 nm. After modification with polydopamine, the particles became rough‐surfaced, spherical‐like nanoparticles with an increased size of ∼ 568 nm. Following polydopamine encapsulation, the zeta potential of the particles changed from ‐6.04 ± 1.18 mV to ‐20.13 ± 6.44 mV (Figure 2c,d). This change indicated alterations in the properties of the particles after polydopamine modification, enhancing their adhesive ability and thereby improving drug retention. Elemental composition analysis of FBZ@BSA@PDA via energy‐dispersive X‐ray spectroscopy (EDS) mapping confirmed the successful loading of BSA and FBZ (Figure S2). UV–vis spectroscopy revealed that FBZ@BSA@PDA exhibited distinct absorption peaks at approximately 280 nm and 330 nm, which was consistent with those of PDA and FBZ@BSA, confirming the successful synthesis of FBZ@BSA@PDA (Figure 2e). Fourier‐transform infrared (FTIR) spectroscopy further revealed characteristic peaks at 3313.3 cm^−^^1^, 1638.1 cm^−^^1^, 1009.7 cm^−^^1^, and 951.2 cm^−^^1^, with notable shifts compared with those of the FBZ@BSA, supporting the effective coating of PDA (Figure 2f). Considering the low pH and high GSH characteristics of the tumor microenvironment, we investigated the release behavior of FBZ@BSA@PDA in solutions with various pH values and GSH concentrations (Figure 2g). The results showed that the release efficiency of FBZ@BSA@PDA significantly increased in a mixed solution of GSH at pH 5.5, reaching its peak release efficiency after approximately 12 h. This finding was consistent with the previous research, and further demonstrated that the system underwent self‐degradation in weakly acidic and high‐GSH conditions. To further evaluate the photothermal conversion efficiency of FBZ@BSA@PDA, we employed a real‐time infrared (IR) thermal imaging system, and the corresponding thermal images demonstrated a significant temperature differential under irradiation. After 8 min of exposure to an 808 nm laser (1.5 W/cm^2^), the temperatures of FBZ@BSA and FBZ@BSA@PDA reached ∼ 23.5 °C and ∼ 55.5 °C, respectively (Figure 2h). These findings confirm the superior photothermal performance of the PDA‐coated system, which is consistent with the IR imaging data (Figure 2i). Moreover, FBZ@BSA@PDA exhibited excellent photothermal stability across four cycles of laser exposure (1.5 W/cm^2^), indicating reliable thermal durability (Figure S3). The photothermal conversion efficiency (η) of FBZ@BSA@PDA was further calculated to be ∼ 26.46%, highlighting its strong potential as an effective photothermal agent.

### 2.2 In Vitro Cytotoxicity and Cellular Uptake of FBZ@BSA@PDA

To elucidate the potential mechanism underlying its antitumor effect, the cellular uptake of the nanoparticles was visualized and quantified. Cy5.5‐labeled FBZ@BSA@PDA was incubated with MB49 cells for 1, 4, and 7 h, followed by confocal laser scanning microscopy (CLSM) observation. Significant internalization into the cytoplasm was observed after 4 h (Figure 2j). Flow cytometry (FCM) analysis (Figure S4) further confirmed that the intracellular fluorescence intensity increased markedly at 4 h and further at 7 h, indicating efficient cellular uptake of FBZ@BSA@PDA, enabling its antitumor activity.

**FIGURE 3:**
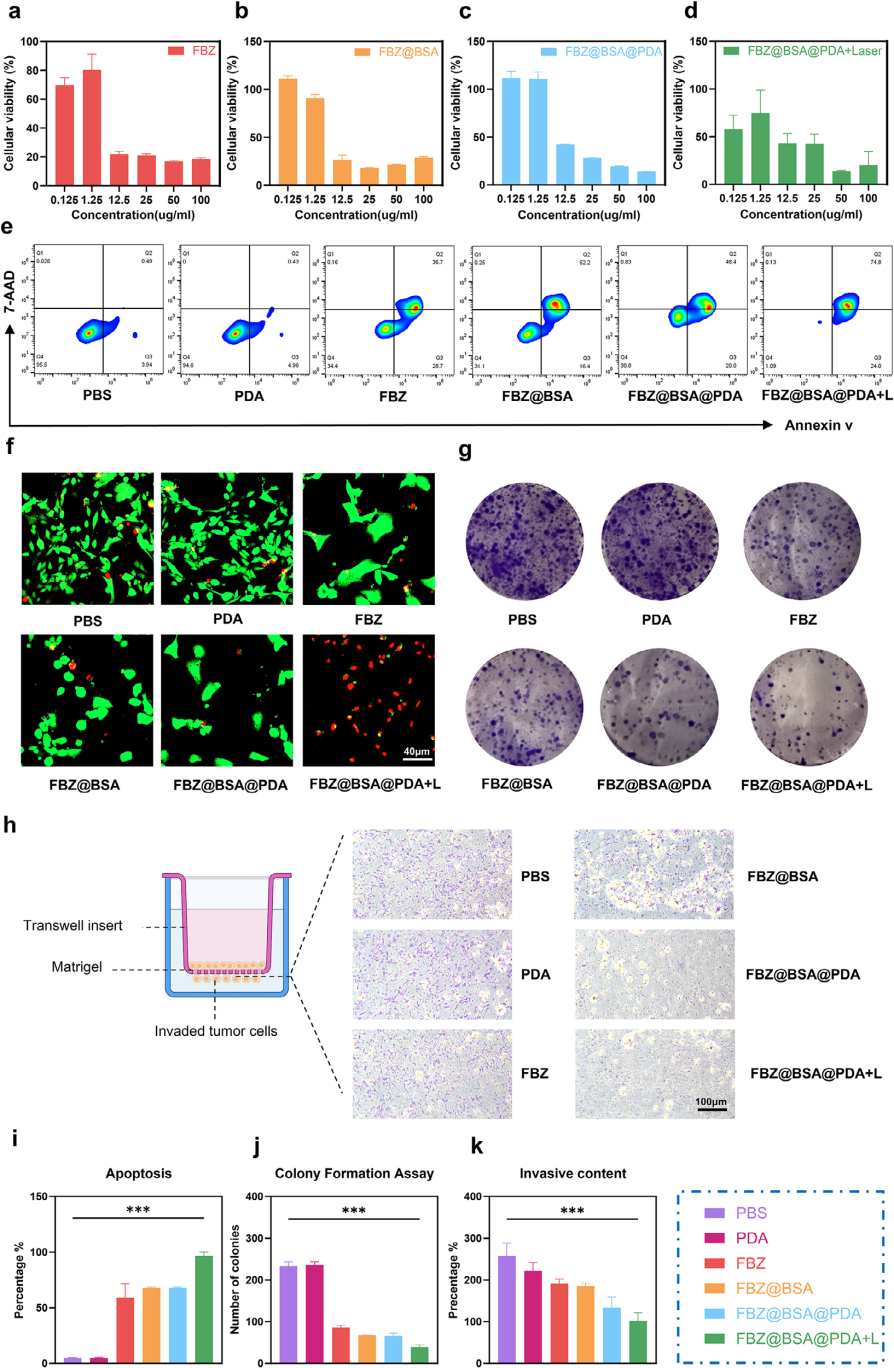
Antitumor activity of FBZ@BSA@PDA in vitro. a–d) Effects of different concentrations of FBZ, FBZ@BSA, FBZ@BSA@PDA, and FBZ@BSA@PDA + laser irradiation on MB49 cell viability assessed via the CCK‐8 assay. e, i) FCM plots and quantitative analysis of apoptosis in MB49 cells across different treatment groups. f) CLSM images of MB49 cells stained with Calcein‐AM (green, viable cells) and PI, (red, dead cells). Scale bar=40 µm. g, j) Crystal violet staining images of MB49 cell migration assays and corresponding semi‐quantitative analysis. h, k) Crystal violet staining images of MB49 colony formation assays and corresponding semi‐quantitative analysis. Scale bar=100 µm * *p* < 0.05; ** *p* < 0.01; *** *p* < 0.001; one‐way analysis of variance (ANOVA).

We further investigated the in vitro therapeutic efficacy of the FBZ@BSA@PDA intravesical delivery system against MB49 cells. Cell viability was assessed via the Cell Counting Kit‐8 (CCK‐8) assay for the PDA, FBZ, FBZ@BSA, FBZ@BSA@PDA, and FBZ@BSA@PDA + 808 nm laser irradiation groups (Figure 3a—d; Figure S5). The PDA nanoparticles exhibited no significant toxicity to cells. Additionally, upon encapsulation of FBZ with BSA and PDA, the toxicity of the resulting formulation remained comparable to that of FBZ alone, which indirectly suggests that the nanoparticles did not exhibit toxicity. Furthermore, following exposure to NIR‐808 nm laser irradiation, the formulation displayed substantial cytotoxicity, even at a low concentration of 1.25 µg/mL. These findings underscore the significant impact of the synergistic effect of PTT in enhancing tumor cell cytotoxicity. Apoptosis assays revealed that FBZ@BSA@PDA under 808 nm laser irradiation resulted in the highest late apoptosis rate (≈74.8%), while no significant difference was observed between the non‐irradiated FBZ@BSA@PDA and FBZ groups, confirming that encapsulation did not impair the antitumor efficacy of FBZ (Figure 3f,j). Live/dead staining with calcein‐AM and PI demonstrated that FBZ@BSA@PDA significantly inhibited tumor cell proliferation, with greater cytotoxicity observed under 808 nm irradiation (Figure 3g). Furthermore, colony formation and cell migration assays (Figure 3h,i,k,l) confirmed that FBZ@BSA@PDA effectively suppressed MB49 cell proliferation and migration. Our results demonstrated that treatment with FBZ@BSA@PDA significantly inhibited both cell proliferation and migration, with no statistically significant difference observed when compared with FBZ alone. These findings suggested that the system had favorable release characteristics and potent anti‐tumor activity. Following PTT, there was a slight improvement in the inhibition of tumor cell proliferation and migration, although the difference was not statistically significant. Above all, while the photothermal effect combined with FBZ@BSA@PDA did not show a marked advantage over FBZ alone in suppressing tumor proliferation and migration, it substantially improved in tumor cell cytotoxicity, demonstrating significant in vitro anti‐tumor efficacy.

### 2.3 RNA‐Seq Analysis Reveals the Mechanism of FBZ@BSA@PDA

**FIGURE 4:**
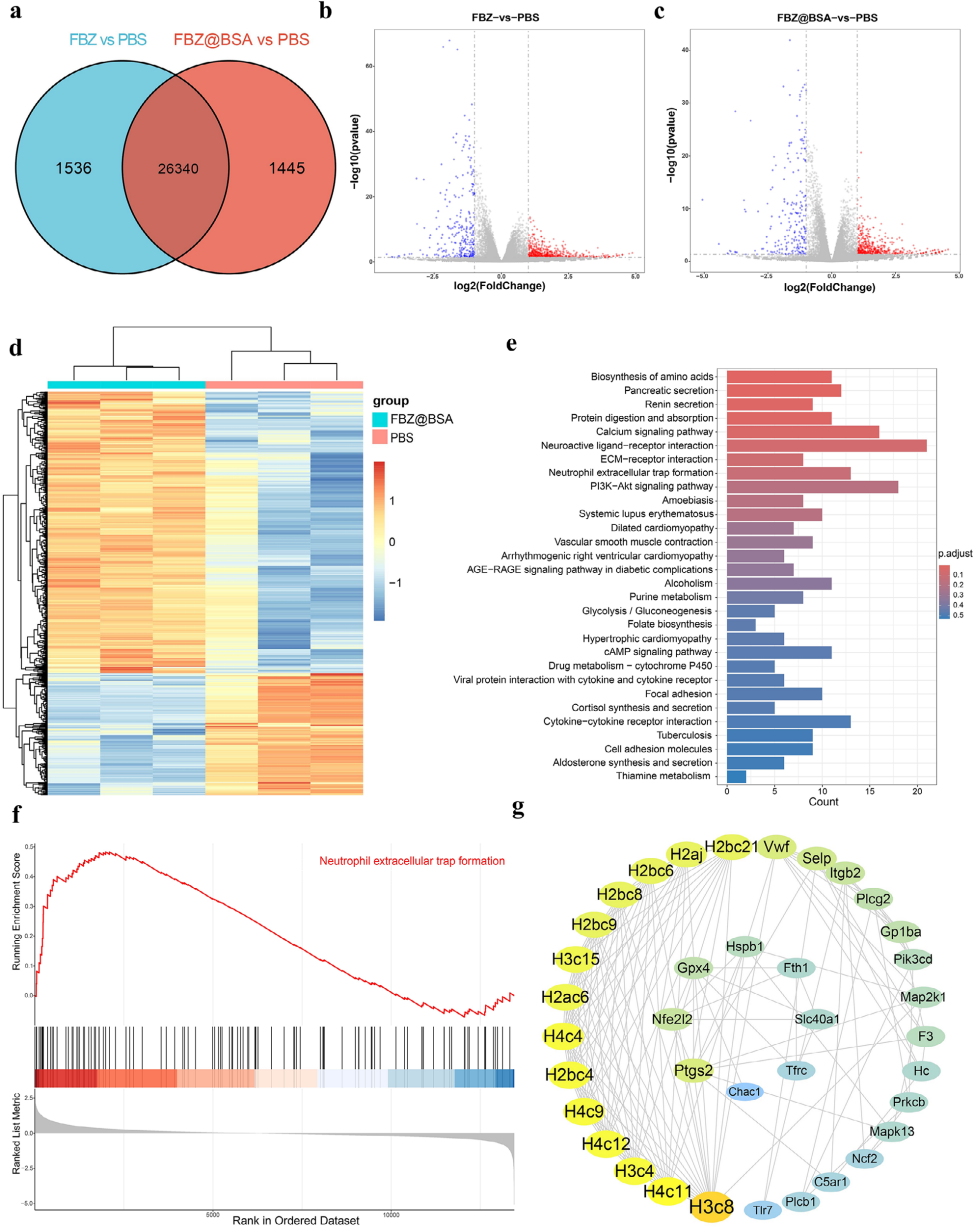
RNA‐seq analysis of MB49 cells treated with FBZ or FBZ@BSA a) Venn diagram revealing the number of genes transcribed in each treatment group. b) Volcano plots displaying the differences between FBZ and PBS‐treated genes, c) FBZ@BSA‐ and PBS‐treated cells. d) Heat‐map of gene expression in cells treated with PBS or FBZ@BSA. e) KEGG analysis of genes differentially expressed between cells treated with PBS or FBZ@BSA. F) GSEA analysis of genes sets associated with neutrophil extracellular trap formation. g) PPI networks of ferroptosis ‐related and huge genes.

To explore the anti‐cancer mechanisms of the perfusion system and consider the potential interference of DMSO, as well as our primary focus on investigating the anti‐cancer mechanism of the core drug FBZ, we treated MB49 cells with FBZ@BSA and FBZ separately and conducted whole‐genome RNA sequencing (RNA‐seq). By comparing the differential genes between the two groups, we identified a total of 26,340 overlapping differential genes, which indirectly suggests that FBZ encapsulated in BSA retains its efficacy. Among the differentially expressed genes, 1,536 were transcribed exclusively in the FBZ‐treated cells, while 1,445 were transcribed solely in the FBZ@BSA‐treated cells. Additionally, compared with PBS treeatment, FBZ treatment upregulated 755 genes (red dots) and downregulated 527 genes (blue dots), while FBZ@BSA treatment led to 884 upregulated genes and 597 downregulated genes. GO and KEGG enrichment analysis revealed that, compared with PBS, FBZ@BSA was involved in mechanisms such as NET formation and the PI3K‐AKT signaling pathway (Figure 4e; Figure S6). GSEA (Gene Set Enrichment Analysis) indicated a significant upregulation of NET formation by FBZ@BSA (Figure 4f).

Although our samples did not contain neutrophils, NET formation is known to involve the generation of ROS and lipid peroxidation (LPO), suggesting a potential association between FBZ@BSA and ferroptosis. Considering the established roles of ROS production and LPO in ferroptosis, the observed NET‐like gene expression profile may represent a manifestation of ferroptosis stress rather than classical neutrophil‐mediated NETosis. This hypothesis aligns with prior findings that linked ferroptosis with dysregulated redox homeostasis and membrane lipid damage. To further validate this possibility, a protein–protein interaction (PPI) network analysis was performed using hub genes identified via our previously established topological network theory [28]. The close interactions between these hub genes and canonical ferroptosis‐related genes provide additional support for the hypothesis that FBZ@BSA exerts anti‐tumor effects, at least in part, through the induction of ferroptosis (Figure 4g). Collectively, these findings indicate that FBZ retains bioactivity after nanoformulation and suggest that ferroptosis‐associated processes may contribute to the anti‐tumor effects of FBZ@BSA. Causal validation will require pharmacological rescue using Ferrostatin‐1 or Liproxstatin‐1, together with quantitative assessment of lipid peroxidation and lipid ROS.

### 2.4 FBZ@BSA@PDA Induced Ferroptosis and Enhanced ICD Via Photothermal Synergy

**FIGURE 5:**
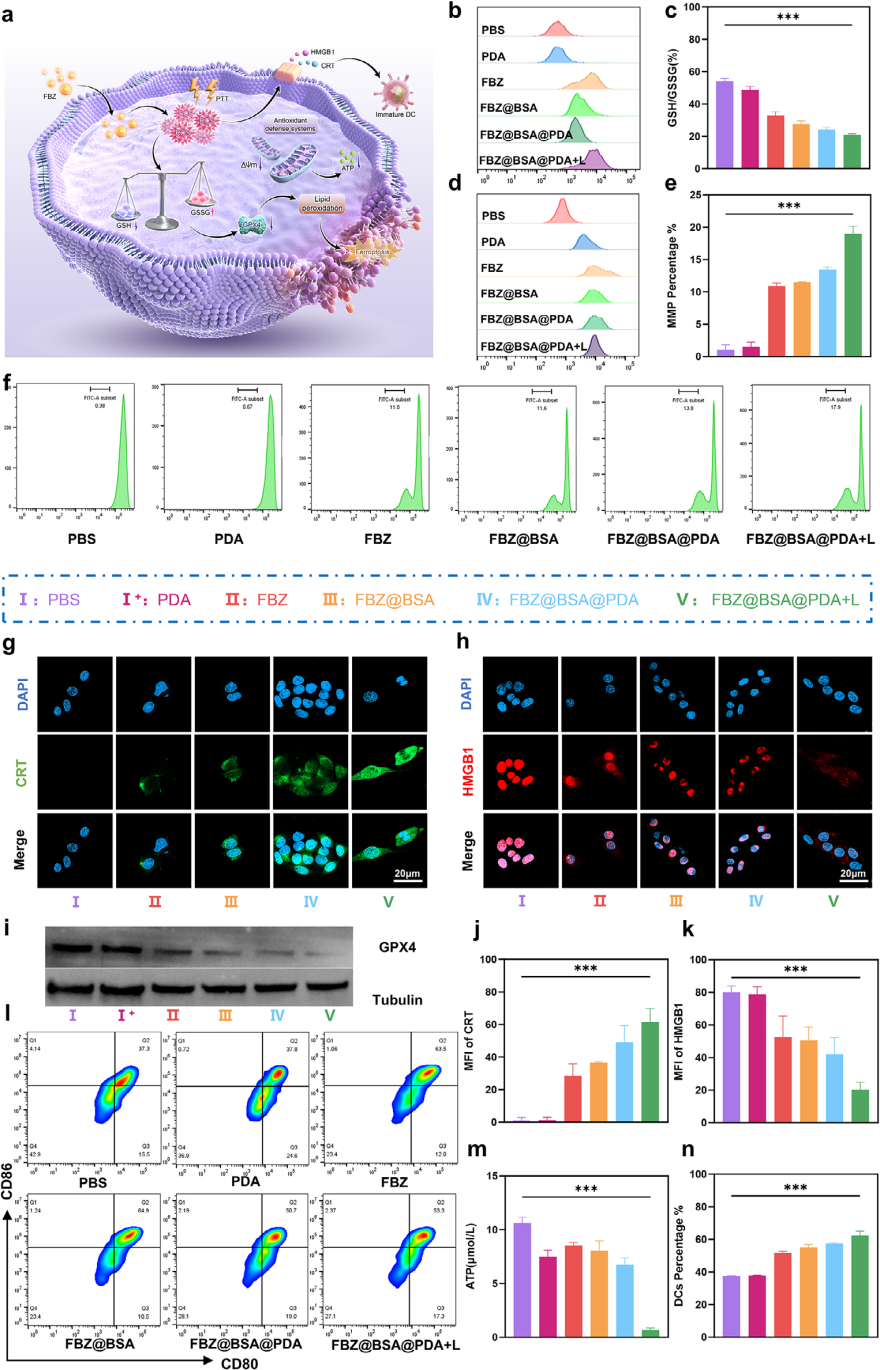
FBZ@BSA@PDA Achieves Antitumor Activity through the Induction of Ferroptosis and ICD in vivo. a) Schematic showing the biological mechanism of ferroptosis and ICD. b) FCM profiles demonstrating the intracellular ROS levels in MB49 cells under different treatments c) The relative GSH/GSSG contents in MB49 cells upon various treatments. c) FCM revealed distinct levels of intracellular LPO in MB49 cells following various treatments. e,f) FCM analysis was conducted to evaluate the effects of different treatments on the mitochondrial membrane potential in MB49 cells. g,j) The CLSM images showing the translocation of CRT to the surface of MB49 cells upon various treatments, accompanied by corresponding fluorescence quantification analysis. Scale bar=20 µm. h,k) CLSM images showing the release of HMGB1 expression upon various treatments of MB49 cells, accompanied by corresponding fluorescence quantification analysis. Scale bar=20 µm. i) The protein expression levels of GPX4 and Tubulin in MB49 cells were determined by Western blotting analysis. l, n) FCM analysis of BMDC maturation following co‐culture with MB49 cells under various treatment conditions. m) The relative ATP contents in MB49 cells after various treatments. (I:PBS. I^+^:PDA. II: FBZ. III: FBZ@BSA. IV: FBZ@BSA@PDA. V: FBZ@BSA@PDA+L). * *p* < 0.05; ** *p* < 0.01; *** *p* < 0.001; one‐way analysis of variance (ANOVA).

RNA‐Seq analysis indicated that FBZ possessed potential ferroptosis‐inducing effects. Consistent with our previous studies, FBZ markedly elevates the levels of ROS, a key mediator of ferroptosis. Mechanistically, Fe^2^^+^‐mediated Fenton reactions produce ROS, leading to downregulation of GPX4, accumulation of LPO, and subsequent cell death [29, 30]. Thus, FBZ‐induced ROS elevation, in combination with photothermal effects, is expected to significantly increase ferroptosis. Unlike other forms of cell death, immunogenic cell death (ICD) is characterized by the explosive release of multiple damage‐associated molecular patterns (DAMPs), such as calreticulin (CRT), which is exposed on the cell surface, the extracellular release of adenosine triphosphate (ATP), and high‐mobility group box 1 (HMGB1), which promotes dendritic cell (DC) maturation and activates antitumor immune responses. In contrast, most DAMPs remain inactive or unexposed during other types of cell death, leading to immune tolerance [23, 31]. This distinction highlights the fundamental difference between ICD and non‐immunogenic forms of cell death. Previous studies have revealed a strong correlation between ferroptosis and ICD induction, where ferroptosis can trigger ICD, while PTT exerts a synergistic enhancing effect [32, 33, 34] (Figure 5a).

To evaluate the ferroptosis‐inducing capability of our perfusion system, we performed flow cytometry to measure the intracellular ROS levels. Compared with those in the PBS and PDA control groups, the ROS generation capacity of the FBZ@BSA@PDA was significantly greater comparable to that of the FBZ alone group, and the ROS levels further increased after combined PTT treatment (Figure 5b). Similarly, intracellular GSH assays showed that FBZ@BSA@PDA, when combined with photothermal treatment, resulted in pronounced GSH depletion, further supporting its ferroptosis‐inducing potential and revealing the synergistic antitumor mechanism of FBZ@BSA@PDA with PTT (Figure 5c). We next analyzed a hallmark of ferroptosis lipid peroxidation (LPO). The results revealed substantial LPO accumulation following FBZ@BSA@PDA pretreatment, which was further enhanced under photothermal irradiation (Figure 5d). These findings corroborated the RNA‐Seq data and indicated that PTT promoted ferroptosis by amplifying ROS production and facilitating lipid peroxidation. Because mitochondrial damage is a crucial indicator of ferroptosis, and mitochondria serve as major sites of ROS generation, we examined the mitochondrial membrane potential (ΔΨm) via flow cytometry. Both the FBZ@BSA@PDA and FBZ treatments led to a decreased ΔΨm, with a more pronounced reduction observed upon photothermal irradiation, which was consistent with our earlier findings that ferroptosis and PTT act synergistically (Figure 5e,f). Western blot analysis further confirmed that all FBZ‐containing groups downregulated GPX4, the core regulatory protein of ferroptosis, and this reduction was more significant under combined photothermal treatment, confirming that PTT facilitates ferroptosis and enhances the therapeutic effect (Figure 5i; Figure S7). To clarify the role of this system in ferroptosis, we intervened with the ferroptosis inhibitor Ferrostatin‐1. We observed that at high concentrations of FBZ@BSA@PDA treatment, no rescue effect was observed. However, as the concentration decreased, there was a noticeable enhancement in cell viability. This suggests that FBZ@BSA@PDA can exert toxic effects via ferroptosis, while also indicating that the system has other anti‐tumor capabilities beyond ferroptosis (Figure 5i; Figure S8). To verify the ability of this system to induce ICD, MB49 cells were subjected to different treatments and then stained for CRT and HMGB1 by immunofluorescence. Increased CRT membrane exposure was observed in both the FBZ@BSA@PDA and FBZ monotherapy groups, indicating that the nanoplatform can induce membrane translocation of CRT. The expression was further elevated under combined photothermal treatment, suggesting a synergistic effect of FBZ@BSA@PDA and PTT in promoting ICD and enhancing immune activation. Moreover, nuclear‐to‐cytoplasmic translocation of HMGB1—another hallmark of ICD—was significantly elevated in both the FBZ@BSA@PDA and FBZ groups, with the most prominent effect observed after photothermal irradiation (Figure 5j,k,m). These results further confirm that FBZ@BSA@PDA effectively induces ICD. ATP assays demonstrated a marked decrease in intracellular ATP following combined FBZ@BSA@PDA and PTT compared with FBZ@BSA@PDA alone, implying enhanced extracellular ATP release. Together, these findings highlight the ability of FBZ@BSA@PDA to induce ferroptosis and enhance ICD under photothermal conditions.

We further co‐cultured the treated tumor cells with DCs. Consistent with previous observations, FBZ treatment promoted DC maturation. Notably, the combination of FBZ@BSA@PDA with PTT markedly enhanced DC maturation, indicating that this synergistic strategy substantially improved immune recognition and antitumor immune responses (Figure 5i; Figure S9). Collectively, these findings suggest that the FBZ@BSA@PDA system, when combined with PTT, not only induces ferroptosis but also amplifies ICD‐mediated immunostimulation, thereby remodeling the tumor immune microenvironment and enhancing overall antitumor efficacy.

### 2.5 Antitumor Efficacy and Biosafety Evaluation of FBZ@BSA@PDA In Vivo

To ensure the applicability of the bladder nano‐instillation system in biological settings, we first evaluated the biosafety of FBZ@BSA@PDA in healthy mice. Eight‐week‐old C57BL/6 mice were intravesically instilled with a single dose of PBS, FBZ@BSA, or FBZ@BSA@PDA, followed by 808 nm laser irradiation. 14 days after treatment, the serum and major organs were collected for analysis. Serum biochemical parameters and hematoxylin and eosin (H&E) staining of major organs showed no significantly different between the treated groups and the PBS control group (Figures S10 and S11), indicating that FBZ@BSA@PDA, with or without photothermal therapy, possesses good biosafety in vivo.

Subsequently, an orthotopic bladder cancer model was established via intravesical implantation of MB49‐luciferase cells to evaluate antitumor efficacy. The tumor‐bearing mice were randomly divided into five treatment groups: PBS, PDA, FBZ@BSA, FBZ@BSA@PDA, and FBZ@BSA@PDA with laser irradiation (808 nm). Dose: FBZ (0.4 mg kg^−1^ per mouse). At Day 0, all mice had comparable tumor burdens, confirmed by bioluminescence imaging. We analyzed the initial fluorescence signal intensity at Day 0 across all groups, and no significant differences in tumor burden were observed (Figure S12). Thus, the tumor loads were consistent among all groups at the start of the experiment, ensuring that any observed treatment effects were not influenced by initial differences in tumor size. Intravesical installation was performed once every three days for a total of four treatment cycles. After each instillation, the urethra was clamped for 1.5 h to ensure maximal bladder drug exposure.

**FIGURE 6:**
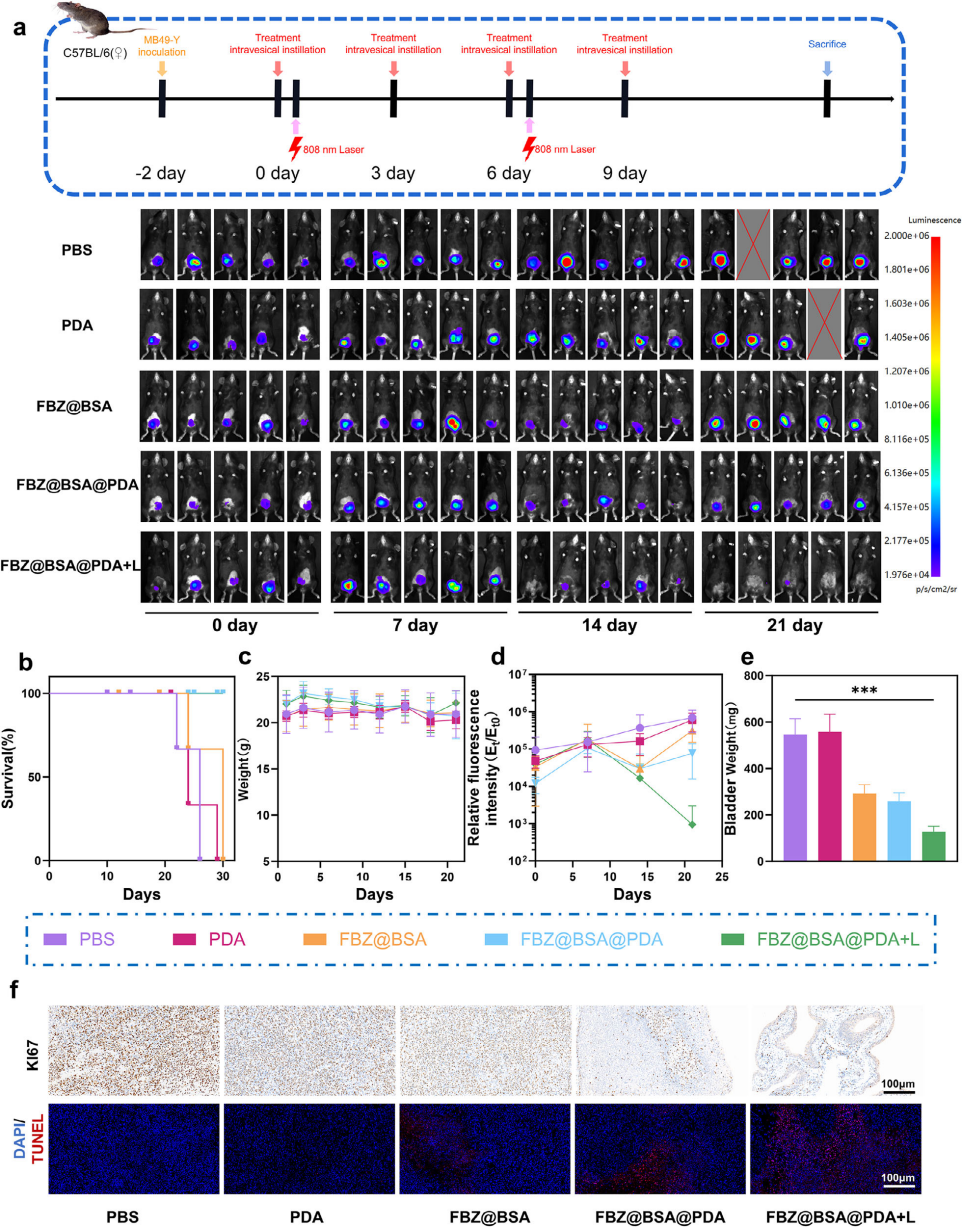
In vivo tumor growth inhibition by FBZ@BSA@PDA. a) Schematic of the treatment schedule and changes in bladder cancer fluorescence intensity during intravesical treatment with PBS, PDA, FBZ@BSA, FBZ@BSA@PDA, or FBZ@BSA@PDA + 808 nm laser. b) Body weight curves of the mice in each treatment group throughout the treatment period. c) Statistical analysis of bladder tumor fluorescence intensity across treatment groups during the treatment cycle. d) Survival curves of the mice in each group at the end of the observation period. e) Statistical analysis of bladder weights from the mice in each group at the end of the observation period. f) Ki‐67 (top) and TUNEL (bottom) staining of tumors extracted from mice treated with different formulations (blue: DAPI; red: TUNEL; scale bar=100 µm). * *p* < 0.05; ** *p* < 0.01; *** *p* < 0.001; one‐way analysis of variance (ANOVA).

In clinical practice, light‐based therapy is administered via intravesical irradiation within the bladder lumen; accordingly, we sought to emulate this treatment modality in mice. Owing to the technical challenges associated with small‐animal intravesical light delivery (including stable transurethral illumination and reproducible intravesical irradiation), photothermal treatment was conducted under aseptic conditions by temporarily exposing the bladder. Specifically, mice were anesthetized, a lower abdominal incision was made, and the bladder was gently exposed and kept moist with sterile saline. An 808 nm laser (1.5 W/cm^2^) was applied for 10 min, while the local temperature was monitored in real time and maintained within the apoptosis‐associated hyperthermia window (43‐50 °C) to achieve efficient tumor killing while minimizing collateral thermal injury. Photothermal irradiation was administered twice, on Day 0 and Day 6, during the treatment regimen. Body weight and bioluminescence imaging were monitored throughout the treatment period (Figure 6a). No significant changes in body weight were detected across the groups (Figure 6b). Notably, the FBZ@BSA@PDA + 808 nm laser group exhibited a marked reduction in tumor bioluminescence after the second treatment, confirming its superior antitumor efficacy (Figure 6c). Subsequently, we evaluated the overall survival of the mice after treatment. Compared with the other groups, both the FBZ@BSA@PDA group and the FBZ@BSA@PDA + 808 nm laser group exhibited markedly improved survival outcomes. Subsequently, we evaluated the overall survival of the mice after treatment and found that both the FBZ@BSA@PDA group and the FBZ@BSA@PDA + 808 nm laser group demonstrated significantly improved survival outcomes compared with the other groups (Figure 6d). At the end of the experiment, the bladders were excised and weighed, and the findings were consistent with previous conclusions, confirming that the FBZ@BSA@PDA + 808 nm laser group had the most pronounced antitumor effect (Figure 6e).

The excised bladder tumors were then sectioned for the subsequent analyses. H&E staining of bladder tissue sections revealed that the FBZ@BSA@PDA + laser group achieved the best therapeutic outcome, with almost no discernible tumor tissue observed in the sections (Figure S13). In contrast, the groups without photothermal treatment showed partial tumor shrinkage, but the overall antitumor efficacy was comparatively limited and less pronounced. Terminal deoxynucleotidyl transferase dUTP nick end labeling (TUNEL) revealed extensive DNA damage (read fluorescence), while Ki‐67 immunostaining indicated reduced tumor cell proliferation in the FBZ@BSA@PDA + Laser group. Collectively, these findings demonstrate that FBZ@BSA@PDA, in combination with PTT, exerts potent in vivo antitumor effects with no systemic toxicity (Figure 6f).

### 2.6 Immune Response Activation Induced by FBZ@BSA@PDA In Vivo

**FIGURE 7:**
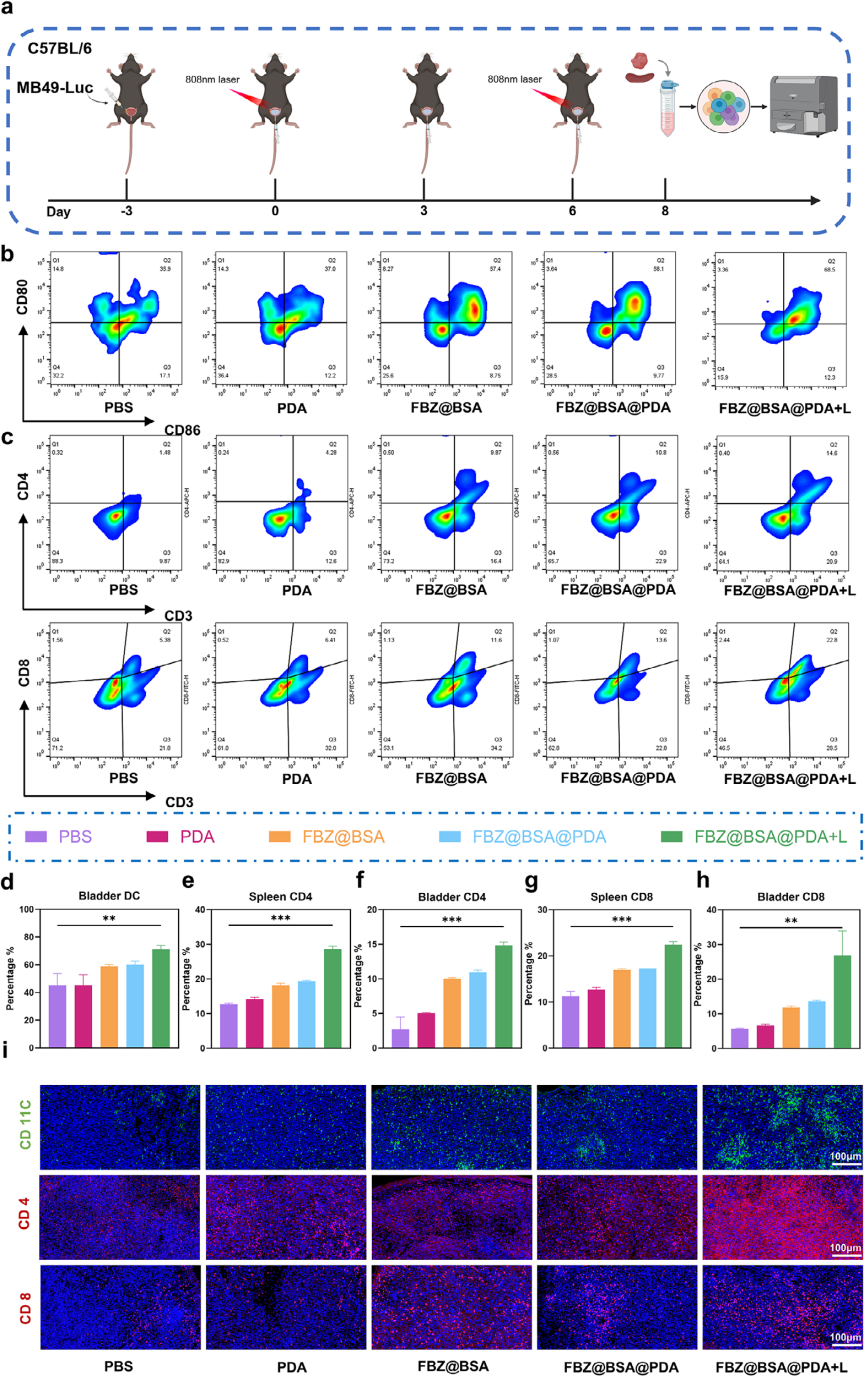
Immune response activation in vivo. a) Schematic of the treatment schedule of immune evaluation during intravesical treatment with PBS, PDA, FBZ@BSA, FBZ@BSA@PDA, or FBZ@BSA@PDA + 808 nm laser. b, d) FCM and statistical analysis of DC activation in the bladder tumors of mice subjected to different treatments. c, g, h) FCM and statistical analysis of CD4^+^ and CD8^+^ T‐cell activation in the bladder tumors of mice treated with different drugs. e, f) Statistical analysis of CD4^+^ and CD8^+^ T‐cell activation in the spleens of mice treated with different drugs. i) Immunofluorescence staining for CD11c, CD4 and CD8 in tumors extracted from mice treated with different drugs (blue, DAPI; green: CD11c; read: CD4/CD8; scale bar=100 µm). * *p* < 0.05; ** *p* < 0.01; *** *p* < 0.001; one‐way analysis of variance (ANOVA).

Building upon the above in vitro findings, we confirmed that FBZ@BSA@PDA, especially when combined with PTT, could induce potent immune activation through ICD and intrinsic material properties. ICD primarily induces immunogenic cell death by promoting DC maturation and subsequently activating T cells. In line with previous studies, we also found that DC activation plays a critical immunostimulatory role in both ferroptosis and ICD [21, 32, 35]. To further assess its in vivo immunostimulatory effects, spleens and bladders from treated mice were collected post‐intravesical instillation, and DC maturation in bladder tissues was analyzed via FCM. The gating strategies for DC cells, CD4^+^ and CD8^+^ T cells are shown in Figure S14. The FBZ@BSA@PDA + 808 nm laser group presented the highest DC maturation rate of 68.5%, markedly surpassing all the other groups (Figure 7b,d). The in vitro DC maturation experiment mentioned above demonstrated that FBZ@BSA@PDA combined with 808 nm irradiation effectively promoted DC maturation, which is consistent with the results of in our in vivo experiments. As a downstream effect of DC activation, T‐cell activation plays an important role in anti‐suit immunity. FCM analysis of the spleen revealed that the percentages of CD4^+^ and CD8^+^ T cells in the FBZ@BSA@PDA + 808 nm laser group reached 28.0% and 23.2% (Figure 7e,f and Figure S15), respectively, which were significantly greater than those in the other groups, indicating that local bladder instillation effectively triggered systemic immune activation. Similar trends were observed in bladder tissues, with CD4^+^ and CD8^+^ T‐cell proportions of 14.6% and 22.8% (Figure 7c,g,h), respectively, in the combined treatment group. Immunofluorescence staining further confirmed the increased infiltration of DCs as well as CD4^+^ and CD8^+^ T cells in the FBZ@BSA@PDA + laser group, which was consistent with the FCM data (Figure 7i). Collectively, these results demonstrate that the novel intravesical drug delivery system can modulate the tumor immune microenvironment, promote DC maturation, and activate effector T cells, thereby potentiating antitumor immunity. Above all, the combination of FBZ@BSA@PDA and PTT effectively activated the immune system, promoted DC maturation, and activated both CD4^+^ and CD8^+^ T cells, highlighting its strong potential in cancer immunotherapy. Overall, the FBZ@BSA@PDA perfusion system, when combined with PTT, induced a robust antitumor immune response and demonstrated exceptional antitumor efficacy.

## 3 Conclusion

In summary, we have developed a novel bladder perfusion system that integrates PTT that elicits ferroptosis‐associated and ICD‐related responses and is accompanied by modulation of the tumor microenvironment, thereby improving therapeutic outcomes in bladder cancer treatment. When combined with PTT, FBZ@BSA@PDA exhibited potent antitumor effects and the ability to induce apoptosis in vitro. Specifically, in the MB49 mouse bladder cancer cell line, FBZ@BSA@PDA in combination with PTT efficiently generated reactive ROS, which in turn induced lipid peroxidation and downregulated the expression of GPX4, a key protein involved in ferroptosis regulation. These results are consistent with the engagement of ferroptosis‐relevant pathways and suggest that a ROS‐driven, lipid peroxidation–dominated process may contribute to the observed cytotoxicity. Moreover, the system also demonstrated ICD‐associated features. Moreover, the system also demonstrated a high potential for ICD induction. By enhancing the exposure of DAMPs, such as CRT and HMGB1, FBZ@BSA@PDA modifies the immune microenvironment, leading to increased immune activation. In addition, FBZ@BSA@PDA effectively induced DC maturation in vitro, further supporting its immune‐stimulating ability. In an orthotopic bladder cancer mouse model, FBZ@BSA@PDA exhibited significant antitumor effects. Notably, when combined with PTT, its therapeutic efficacy was markedly enhanced. In vivo immune evaluations revealed robust immune activation, which was consistent with the findings from the in vitro experiments. Taken together, FBZ@BSA@PDA represents a promising intravesical perfusion system that provides a localized therapeutic strategy for bladder cancer by combining PTT with ferroptosis‐associated oxidative injury, ICD‐related immunogenic signals, and consequent immune activation.

## Author Contributions

X.X., R.L., A.Z. and J.Z. contributed equally to this work and are joint first authors. J.H., L.C., and S.Z. were responsible for the design and improvement of the project. X.X., R.L., A.Z. and J.Z. conducted all the experimental work, including the factual experiments and their replication. R.L.and Y.L. prepared the figures and performed data visualization. D.D., Q.Z. and L.L provided guidance and optimization for the experiments. L.P., M.L. and Y.H. contributed to data analysis and interpretation. All authors reviewed the manuscript and approved its submission.

## Ethical Statement

All procedures involving animals were carried out in full compliance with the applicable guidelines and regulations for the care and use of laboratory animals. The study protocol received formal approval from both the Experimental Committee and the Ethics Committee of Affiliated Luohu Hospital of Shenzhen University (Approval No.: IACUC‐202300167).

## Conflicts of Interest

The authors declare no conflicts of interest.

## Supporting information




**Supporting File**: advs74876‐sup‐0001‐SuppMat.docx.

## Data Availability

The data that support the findings of this study are available from the corresponding author upon reasonable request.
