# Supplementary material for: Ferroptosis Induction by Fenbendazole Combined With Photothermal Therapy Triggers Dual‐Immunotherapy Against Bladder Cancer
Source: Adv Sci (Weinh). 2026 Mar 20;13(31):e74876. doi: 10.1002/advs.74876 (PMC13587987; doi:10.1002/advs.74876)
Supplement: Supplementary file 1 [file ADVS-13-e74876-s001.docx]

**Supporting Information**

**Ferroptosis Induction by Fenbendazole Combined with Photothermal Therapy Triggers Dual Immunotherapy Against Bladder Cancer**

Xiaojian Xu^a#^, Rui Liang^c＃^, Anguo Zhao^b#^, Jun Zhang^a＃^, Rongkang Li^c^, Yang Liu^c^, Dashi Deng^d^, Qi Zhuang^c^, Lisha Liu^d^, Lei Peng^c^, Miao Li^a^, Xuedong Wei ^a^, Yuhua Huang^a^, Shaohua Zhang^c^*,

Liang Cheng^e^*, and Jianquan Hou^a,b^*

^a^ Department of Urology, The First Affiliated Hospital of Soochow University,

Suzhou 215000 China.

^b^ Department of Urology, The Fourth Affiliated Hospital of Soochow University, Medical Center of Soochow University, Suzhou Dushu Lake Hospital, Suzhou 215000 China.

^c^ Department of Urology, South China Hospital, Medical School, Shenzhen University,

Shenzhen, 518116, China

^d^ Department of Urology, The Affiliated Luohu Hospital of Shenzhen University, Shenzhen University, Shenzhen, 518001, China.

^e^ Institute of Functional Nano & Soft Materials (FUNSOM), Jiangsu Key Laboratory for Carbon-Based Functional Materials & Devices, Soochow University, Suzhou, 215123, China

*^#^These authors contributed equally to this work and are joint first authors.*

*Corresponding authors: Jianquan Hou, Liang Cheng, Shaohua Zhang

**Emails:** houjianquan@suda.edu.cn; [lcheng2@suda.edu.cn](mailto:lcheng2@suda.edu.cn); zhangshaohua@szu.edu.cn

**Materials and methods**

**Materials and reagents**

Dopamine hydrochloride was purchased from Merck (Darmstadt, Germany). Fenbendazole (FBZ) was obtained from Aladdin (Shanghai, China). Dimethyl sulfoxide (DMSO) and bovine serum albumin (BSA) were supplied by Solarbio (Beijing, China). A Cell Counting Kit-8 (CCK-8) was purchased from ZETA Life (San Francisco, USA). RPMI-1640 and Dulbecco’s Modified Eagle Medium (DMEM), 0.25% trypsin–EDTA, fetal bovine serum (FBS), and penicillin–streptomycin (P/S) were purchased from Gibco (Grand Island, USA). 4′,6-Diamidino-2-phenylindole (DAPI) was obtained from UNIV-Bio (Shanghai, China). Antibodies, including FITC-anti-mouse CD80, APC-anti-mouse CD86, PE-anti-mouse CD3, FITC-anti-mouse CD8, and APC-anti-mouse CD4, were obtained from BioLegend (San Diego, USA).

**Cell lines and animals**

Murine bladder cancer MB49 and MB49-luciferase (MB49-Luc) cells were cultured in high-glucose DMEM supplemented with 10% FBS and 100 U/mL penicillin–streptomycin at 37 °C in a humidified incubator containing 5% CO₂. Eight-week-old female C57BL/6 mice were purchased from Zhiyuan Biotechnology Co., Ltd. (Guangzhou, China), and all animal procedures were approved and supervised by the Institutional Animal Care and Use Committee of Luohu Hospital of Shenzhen University.

**Synthesis of FBZ@BSA@PDA nanocomposites**

**Preparation of FBZ@BSA:** FBZ (1.2 mg) was dissolved in DMSO (100 μL), and BSA (6.6 mg) was dissolved in ultrapure water. The BSA solution was preheated to 75 °C for 15 min, followed by mixing with FBZ solution under stirring on ice for 1 h. The mixture was purified by centrifugation (4000 rpm, 20 min) and lyophilized to obtain FBZ@BSA powder.

**PDA coating:** Dopamine (5 mg) and FBZ@BSA were dispersed in Tris–HCl buffer (pH= 8.5) and shaken at 37 °C for 30 min to simulate in situ polymerization in the bladder, yielding FBZ@BSA@PDA nanocomposites.

**Synthesis of FBZ@BSA@PDA-Cy5.5**

FBZ@BSA@PDA was reacted with EDC (25 mg) and NHS-Cy5.5 (0.01 mg) in deionized water at 4 °C overnight. The product was collected by centrifugation (14800 rpm, 20 min) and denoted as FBZ@BSA@PDA-Cy5.5.

**Cellular Uptake Analysis**

MB49 cells (8×10⁴ cells/mL) were seeded onto glass coverslips in 24-well plates and incubated for 12 h, followed by treatment with FBZ@BSA@PDA-Cy5.5 for 1, 4, or 7 h. The cells were fixed with 4% paraformaldehyde, the nuclei were stained with DAPI, and the cytoskeleton was labeled with Actin-Tracker Green-488. Images were captured via CLSM (LSM-800, Carl Zeiss, Germany). MB49 cells (3×10⁵ cells/well) were seeded in 12-well plates, treated identically, and analyzed via FCM.

**Cytotoxicity Assay**

Cell viability was evaluated via the Cell Counting Kit-8 (CCK-8) assay. MB49 cells (8×10³ cells/well) were treated with PDA, FBZ, FBZ@BSA, FBZ@BSA@PDA, or FBZ@BSA@PDA+NIR (808 nm, 1.5 W/cm², 10 min) for 24 h. CCK-8 reagent was added, the mixture was incubated for 2 h at 37 °C, and the absorbance was measured at 450 nm.

**Apoptosis analysis**

MB49 cells (3×10⁵ cells/well) were treated as described above, stained with an annexin V-APC/7-AAD apoptosis detection kit (Elabscience, China), and analyzed via FCM.

**Colony formation and migration assays**

MB49 cells (2000 cells/well) were seeded in 6-well plates, cultured for 5 days, treated for 24 h, and further cultured for 3 days before fixation and crystal violet staining. MB49 cells (3×10⁵ cells/well) were seeded in 12-well Transwell chambers, treated for 24 h, fixed, and stained with crystal violet for microscopic analysis.

**ROS, GSH/GSSG, and LPO detection**

**ROS:** MB49 cells (3×10⁵ cells/well) were seeded in 12-well chambers, treated for 24 h, incubated with DCFH-DA for 20 min, and analyzed via FCM.

**GSH/GSSG:** MB49 cells (1×10^6^ cells/well) were seeded in 6-well chambers, the cell lysates were collected, and the GSH/GSSG ratios were quantified using a microplate reader.

**LPO:** MB49 cells (1×10^6^ cells/well) were seeded in 6-well chambers,BODIPY 581/591 C11 was used to stain the cells for 30 min, and lipid peroxidation was analyzed via FCM.

**ATP release, CRT exposure, and HMGB1 translocation**

After treating MB49-Luc bladder cancer cells with the corresponding formulations and incubating them for 24 h, the culture supernatants were collected, and extracellular ATP levels were quantified using a luminescence-based ATP detection kit to assess treatment-induced immunogenic cell death (ICD). Subsequently, the treated MB49-Luc cells were subjected to immunofluorescence staining, and the exposure of CRT on the cell surface as well as the nuclear-to-cytoplasmic translocation of HMGB1 were examined using confocal laser scanning microscopy to further confirm the activation of ICD-related signals.

**In vitro DC maturation**

BMDCs were derived from the bone marrow of 4-week-old C57BL/6 mice and cultured with GM-CSF (20 ng/mL) and IL-4 (10 ng/mL) for 5 days. BMDCs were co-cultured with different formulations for 24 h, stained with CD11c-PE, CD80-FITC, and CD86-APC, and analyzed via FCM.

**Western blotting analysis**

After the cells were incubated with the treatments, the total proteins were extracted via RIPA buffer containing protease and phosphatase inhibitors, quantified via a BCA assay, separated via SDS–PAGE, transferred to PVDF membranes, incubated with primary antibodies (e.g., GPX4), and detected with HRP-conjugated secondary antibodies via enhanced chemiluminescent (ECL) substrate.

**Establishment of orthotopic bladder cancer model**

MB49-Luc cells (5×10⁵ cells in 20 μL of PBS) were injected into the bladder wall of anesthetized C57BL/6 mice. Successful inoculation was confirmed by in vivo bioluminescence imaging at day 3 post-inoculation.

**In vivo antitumor efficacy and immune activation**

The mice were randomized into five groups and treated intravesically with PBS, PDA, FBZ@BSA, FBZ@BSA@PDA, or FBZ@BSA@PDA+NIR every 3 days for 4 cycles. Tumor growth was monitored via bioluminescence imaging, and body weights were recorded. At the end of treatment, the bladders were excised and weighed. Spleen and bladder tissues were collected for FCM analysis of DC maturation and CD4/CD8 T-cell populations.

**Statistical analysis**

Data analysis was conducted using GraphPad Prism version 10.0. For comparisons between two groups, an unpaired t-test was applied, while differences among three or more groups were evaluated using one-way ANOVA. A p-value of less than 0.05 was considered statistically significant. Significance was denoted as follows: * p < 0.05, ** p < 0.01 and *** p < 0.001. Bar charts illustrating the results were also generated in GraphPad Prism 10.0.

**Figure S1.**TEM images of FBZ@BSA (a) and FBZ@BSA@PDA(b)

**Figure S2.** Elemental spectrograms of FBZ@BSA@PDA were analyzed using energy dispersive X-ray spectroscopy (EDS) mapping.

**Figure S3**. Thermal images of FBZ@BSA@PDA under 1.5 W cm⁻² irradiation, captured every 20 s

**Figure S4**. FCM image showing the uptake of FBZ@BSA@PDA-Cy5.5 by MB49 cells after 1, 4, and 7 h of co-incubation.

**Figure S5**. Effects of different concentrations of PDA on MB49 cell viability, as assessed via the Cell Counting Kit-8 (CCK-8) assay.

**Figure S6.** GO analysis of differentially expressed genes between cells treated with PBS or FBZ@BSA.

**Figure S7.** Relative grey value of the ferroptosis-related protein GPX4.

**Figure S8.** Cell viability curve of MB49 tumor cells after Ferrostatin-1 intervention.

**Figure S9.** FCM analysis of BMDC maturation following co-culture with MB49 cells under LPS.

**Figure S10**. The serum hepatic and renal function indices of the mice with different treatments were analyzed. a) alanine aminotransferase (ALT), b) aspartate aminotransferase (AST), c) γ-glutamyl aminotransferase (γ-GT), d) blood urea nitrogen (BUN), e) and uric acid (UA), f) creatinine (CREA).

**Figure S11.** HE-stained sections of the heart, liver, spleen, lungs, kidneys and bladder of the mice with different treatments (PBS, PDA, FBZ@BSA, FBZ@BSA@PDA, or FBZ@BSA@PDA+Laser). Scale bar=50 μm.

**Figure S12.** Fluorescence values of tumors in mice from different groups on day 0.

**Figure S13.** H&E images of mouse bladder tumors of the mice with different treatments (PBS, PDA, FBZ@BSA, FBZ@BSA@PDA, or FBZ@BSA@PDA+Laser). Scale bar=500 μm.

**Figure S14.** FCM gating strategies for the identification of DC(a), CD4^+^ and CD8^+^T cell (b).

**Figure S15.** FCM of CD4^+^ and CD8^+^ T cell activation in the spleens of mice treated with different drugs.
